# Targeting apoptosis in acute myeloid leukaemia

**DOI:** 10.1038/bjc.2017.281

**Published:** 2017-08-24

**Authors:** Philippe A Cassier, Marie Castets, Amine Belhabri, Norbert Vey

**Affiliations:** 1 Department of Medical Oncology, Centre Léon Bérard, 69008 Lyon, France; 2 Centre Léon Bérard, Centre de Recherche en Cancérologie de Lyon, 69008 Lyon, France; 3 Department of Hematology, Centre Léon Bérard, 69008 Lyon, France; 4 Department of Hematology, Institut Paoli-Calmettes, 13009 Marseille, France

**Keywords:** apoptosis, acute myeloid leukaemia, BCL2, MDM2, XIAP

## Abstract

Acute myeloid leukaemia (AML) is a molecularly and clinically heterogeneous disease, and its incidence is increasing as the populations in Western countries age. Despite major advances in understanding the genetic landscape of AML and its impact on the biology of the disease, standard therapy has not changed significantly in the last three decades. Allogeneic haematopoietic stem cell transplantation remains the best chance for cure, but can only be offered to a minority of younger fit patients. Molecularly targeted drugs aiming at restoring apoptosis in leukaemic cells have shown encouraging activity in early clinical trials and some of these drugs are currently being evaluated in randomised controlled trials. In this review, we discuss the current development of drugs designed to trigger cell death in AML.

Acute myeloid leukaemia (AML) is characterised by the rapid proliferation of immature myeloid progenitors resulting in the suppression of normal haematopoiesis. Standard therapy includes intensive chemotherapy, followed in patients with poor prognostic features by high dose chemotherapy followed by allogeneic bone marrow transplant (Ferrara and Schiffer, 2013). However, because of the associated morbidity, intensive chemotherapy followed by allogeneic transplant is only feasible in young patients. From an epidemiological perspective however, AML affects primarily patients older than 60 years, who are not eligible for such treatments whereas their prognostic is overall poor without long-term survival (Ossenkoppele and Löwenberg, 2015). There is therefore an unmet medical need to develop new therapies for AML patients.

From the molecular point of view, AML is thought to develop through a multistep process progressing through the acquisition by tumour cells of multiple genomic alterations, which affect several cellular parameters. Genomic alterations in AML have classically been classified in those inducing a block in differentiation, those inducing proliferation and more recently those affecting epigenetic control (Döhner *et al*, 2010; Fathi and Abdel-Wahab, 2012). This classification has recently been challenged by data suggesting that alterations do not happen independently of each other but rather cooperate in disease progression (Shih *et al*, 2015).

Apoptosis is one of the mechanisms of regulated cell death and has been the focus of intensive research over the last century. Evasion from apoptosis is a required step for malignant tumour progression (Hanahan and Weinberg, 2011). Apoptosis is accomplished through two separate but connected pathways: the intrinsic pathway which converges on the mitochondria and leads to the formation of the apoptosome and caspase-9 activation; and the extrinsic pathway which transduces signalling from external apoptotic cues (see Figure 1).

Activation of the intrinsic pathway is under the control of the BCL2 family of protein, which can be divided into anti-apoptotic proteins (BCL2, BCL-XL, BCL-W (BCL2L2), myeloid cell leukaemia sequence 1 (MCL1) et BFL1/A1 (BCL2A1)), pro-apoptotic BH3 only proteins (BH3-interacting domain death agonist (BID), BCL-2 antagonist of cell death (BAD), BCL-2-interacting killer (BIK), PUMA (BCL-2-binding component 3 (BBC3)), NOXA (phorbol-12-myristate-13-acetate-induced protein 1 (PMAIP1)), BCL2-modifying factor (BMF) and HRK) and pro-apoptotic effector proteins (BCL-2-associated X protein (BAX), BCL-2-antagonist/killer (BAK) and BCL-2-related ovarian killer (BOK)). Upon apoptotic stimuli, BH3-only proteins are upregulated (e.g. PUMA and NOXA upon p53 activation), and anti-apoptotic BCL2 family proteins are downregulated, leading to change in the balance of pro- *vs* anti-apoptotic BCL2 family proteins. Other BH-3 only proteins can be induced by various stimuli, such as BIM which is stabilised in response to E2F1. This unbalance leads to activation of the effector proteins BAK and BAX, which assemble into multimeric pores in the mitochondrial membrane, lead to mitochondrial outer membrane permeabilisation and cytochrome c release into the cytosol (Ashkenazi *et al*, 2017). The release of cytochrome c leads to the formation of the apoptosome via recruitment of APAF1 and pro-caspase-9, followed by activation of caspase-9 by proteolytic cleavage. Caspase-9 activates caspase-3 and -7 by proteolysis.

The extrinsic pathway comprises the death receptors DR4 and DR5 as well as FAS, which are members of the TNF-receptor family, and share the common trait of having a death domain. This allows the formation of a death-inducing signalling complex by recruiting FAS-associated Death Domain and pro-caspase-8, which leads to the proteolytic activation of caspase-8, which in turn activates caspase-3 and -7. The extrinsic pathway is regulated by several inhibitor of apoptosis proteins (IAPs) which inhibit the transmission of the apoptotic signal at different levels. Eight IAP family members have been identified in humans: XIAP (X-linked inhibitor of apoptosis), cellular inhibitor of apoptosis 1 (cIAP-1), cIAP-2, survivin, NAIP (neuronal apoptosis inhibitory protein), livin, BRUCE/Apollon and IAP-like protein 2. Their grouping as a family is linked to the presence of 1–3 baculovirus IAP repeats (BIR) domain. XIAP cIAP1 (BIRC2), cIAP2 (BIRC3) are the sole members of the family functioning as inhibitors of apoptosis and XIAP is the only direct inhibitor of caspases 3, 7 and 9. Cellular IAP (cIAP) 1 and 2 inhibit caspases by targeting them for ubiquitin-mediated proteasomal degradation which as consequences in apoptosis but also other signalling events: for example, modulation of caspase 8 stability influences the transmission of apoptotic signals by the death-inducing signalling complex, but also modulates the NF-κB survival pathway. Several naturally occurring inhibitors of IAP have been identified, such as Smac/DIABLO (second mitochondrial-derived activator of caspases/direct IAP binding protein with low PI), Omi/HrtA2 (HTRA serine peptidase 2) and XAF-1 (XIAP-associated factor 1), but among these, Smac/DIABLO appears to be the strongest IAP inhibitor (Obexer and Ausserlechner, 2014).

Aside from the apoptotic machinery, another protein that plays a central role in regulating apoptosis is the p53 protein, which integrates a number of signals resulting from various cellular insults and induces adequate cellular response by inducing cell-cycle arrest, repair and/or apoptosis. Termed the ‘guardian of the genome’, p53 regulates cell fate following several types of DNA insults. Two different outcomes can be mediated by activated p53: cell cycle arrest (and DNA repair) or apoptosis (if DNA repair is not possible). Cell cycle arrest following p53 activation is mediated by p21 (WAF1/CIP1, coded by the tumour suppressor gene *CDKN1A*), while PUMA, NOXA and BIM are the main mediators of p53-induced apoptosis (Yu *et al*, 2001; Villunger *et al*, 2003; Schlereth *et al*, 2010).

Identifying strategies to induce or restore defective apoptosis is therefore a high priority challenge for cancer therapy (Fesik, 2005). The apoptotic pathways can be therapeutically targeted at several levels, which can be grouped into two broad mechanisms: (i) induction of apoptosis, for example by restoring active p53, or by activating death receptors or (ii) restoration of downstream signalling cascades. Unlike many solid tumours, in which one of the most common events leading to a block in apoptosis is a genomic loss of p53 by either deletion or mutation, the *TP53* locus is wild-type in most cases of non-complex karyotype *de novo* AML (Haferlach *et al*, 2008; Rücker *et al*, 2012; Cancer Genome Atlas Research Network, 2013), suggesting that alternate mechanisms are at play to block apoptosis in the majority of AML cases.

In this review, we discuss alterations in apoptosis cascades underlying AML and the current development of drugs designed to trigger cell death in these malignancies.

## Mechanisms of dysregulation of apoptosis in AML

Apoptosis is dysregulated in virtually all malignancies but the means of dysregulation and the signalling elements involved are highly variable from one tumour type to another. For example, although the tumour suppressor *TP53* is the most commonly mutated gene in human solid tumours, genomic inactivation of *TP53* is much less common in haematological malignancies (Hainaut and Pfeifer, 2016). When interrogating the cbioportal.org repository (www.cBioportal.org– Cerami *et al*, 2012; Gao *et al*, 2013), *TP53* gene alterations are found in 2.8–10.6% of adult leukaemia and in about 9% of AML cases (Cancer Genome Atlas Research Network, 2013). Interestingly, *TP53* alterations in AML are associated with distinct genomic and biological characteristics, such as complex karyotype and increased genomic instability, which correlate with poor prognosis (Haferlah *et al*, 2008; Rücker *et al*, 2012). This observation has led to the identification of ‘AML with *TP53* mutations, chromosomal aneuploidy, or both’ as a separate prognostic subgroup, encompassing the previously identified subgroup of patients with complex karyotype AML (Papaemanuil *et al*, 2016). While most commonly associated with resistance to chemotherapy, *TP53* alterations were recently shown to be associated with improved response rate in patients treated with decitabine (Welch *et al*, 2016). Interestingly, in this study, survival was similar in patient with poor risk cytogenetic features/*TP53* mutation and patients with intermediate-risk cytogenetics/wild-type *TP53*.

The frequency of *TP53* gene alterations remains low in *de novo* and/or non-complex karyotype AML. However, functional inactivation of p53 or of its pathway appears to be a requisite for transformation; loss of p53 function in cancer cells with wild-type *TP53* is often caused by abnormalities in p53-regulatory proteins, including overexpression of mouse double minute 2 (MDM2)/MDMX, deletion of *CDKN2A/ARF*, and alterations in *ATM*. Contrary to solid tumours, the *CDKN2A* locus and *ATM* are rarely altered in AML. Likewise, MDM2 amplification is rare in AML, but its overexpression has been shown in several studies and correlates with shorter progression-free survival (Faderl *et al*, 2000). MDM2 overexpression also correlates with wild-type *TP53* gene status and loss of p21WAF1/CIP1 expression (Quintás-Cardama *et al*, 2017) which supports its pathological implication as a mean to escape apoptosis.

Downstream effectors of the intrinsic apoptotic pathway are also deregulated in AML, but similar to the case of MDM2, genomic alterations are rare. This may be explained by a founding role of genomic alteration of epigenetic regulators in AML (Papemmanuil *et al*, 2016) which may lead to deregulated expression without genetic alteration. Thus, BCL2 and other members of the BCL2 family of proteins have been reported to be overexpressed in AML and correlate with resistance to cytotoxic chemotherapy and to targeted agents (Kornblau *et al*, 1999; Mehta *et al*, 2013). More recently, overexpression of BCL-XL (*BCL2L1*) and MCL1 were shown to also play a crucial role in AML pathogenesis (Xiang *et al*, 2010; Glaser *et al*, 2012).

The role of the extrinsic apoptotic pathway in AML appears less clear. Here again no recurrent genetic alterations have been reported, but leukaemic blasts were shown to decrease or lose FAS expression during transformation from myelodysplastic syndrome to AML and this correlates with increased methylation of the FAS promoter. Treatment with decitabine was shown to induce FAS expression as well as DR5 expression and was suggested to be one of the mechanisms of action of demethylating agents (Ettou *et al*, 2013; Karlic *et al*, 2014). IAPs are also important mediators of resistance to therapy in AML. IAP proteins have been shown to be expressed in samples from AML patients and higher expression correlated with lack of complete response (CR) following induction with standard (intensive) chemotherapy (Pluta *et al*, 2015).

## Targeting the MDM2/p53 pathway

MDM2 regulates p53 stability via ubiquitination, which promotes p53 degradation by the proteasome. MDMX on the contrary has no intrinsic ubiquitin ligase activity and may act on p53 by heterodimerising with MDM2, thereby enhancing its ubiquitin ligase activity. The regulation of MDM2 and MDMX levels is complex but their expressions are increased by oncogenic signals. It should be noted that both of these genes are direct p53 transcriptional targets, though MDM2 is more broadly responsive to p53 activation (Phillips *et al*, 2010). The discovery of the nutlin family of compounds, which are small molecules able to disrupt the MDM2-p53 interaction, by Vassilev *et al* in 2004 (Vassilev *et al*, 2004) opened the way for efficiently targeting this pathway in the clinic. Other classes of agents have since then been shown to be able to disrupt this interaction (Ding *et al*, 2006) and several MDM2 antagonists are currently in clinical development in solid tumours and haematological malignancies. These compounds induce p53 stabilisation and induction of its target genes (*CDKN1A* and *PUMA* among others). Many studies have shown the efficacy of MDM2 inhibitors in preclinical models of AML, either alone or combined with various inhibitors, including those targeting the mitogen-activated protein kinase pathway (Long *et al*, 2010; Zang *et al*, 2010; Weisberg *et al*, 2015; Lehmann *et al*, 2016). These preclinical studies have shown that approximately 50–60% of AML cell lines and patients-derived AML samples are sensitive to MDM2 inhibition (Long *et al*, 2010; Weisberg *et al*, 2015). As expected given the mode of action of MDM2 inhibitors, TP53 mutated cells are intrinsically resistant to this approach.

The available clinical data regarding MDM2 inhibitors in AML is currently limited to phase 1 trial data. The first MDM2 antagonist used in the clinic was RG7112 (Roche, NJ, USA), a nutlin derivative given orally. The recently published phase 1 study of RG7112 in patients with leukaemia showed that this agent had meaningful clinical activity in AML, but not in other types of leukaemia, with 5 of 30 evaluable AML patients having an International Working Group (IWG)-defined response (Andreeff *et al*, 2016). Interestingly, two patients with *TP53*-mutant AML displayed transient clinical response. The main adverse events were gastrointestinal toxicity, comprising nausea, vomiting and diarrhoea, which were dose-limiting and bone marrow suppression resulting in neutropenia, febrile neutropenia and thrombocytopenia. The maximum tolerated dose of RG7112 in patients with AML was declared at 1500 mg BID for the first 10 days of 28-day cycles. Pharmacokinetic analysis in this study showed that the mean exposure at MTD was slightly lower than the expected efficacious exposure in animal models. This prompted Roche to develop a second generation, more potent MDM2 inhibitor (RG7388–idasanutlin), which has been tested as an orally given drug in patients with solid tumours and as an oral and intravenous formulation in patients with AML. In these studies, the safety profile of RG7388 was in fact comparable to that of RG7112, suggesting that the observed adverse events result from on-target effects on the gastrointestinal tract and the bone marrow (Siu *et al*, 2014). The safety profile is consistent across several pharmacological classes of MDM2 inhibitors tested in the clinic (Kurzrock *et al*, 2012; Wagner *et al*, 2015), confirming on-target effects. The use of an intravenous formulation limited the impact of gastrointestinal toxicity on dosing and exposure, and interesting results have been reported with idasanutlin intravenously administered with cytarabine (Yee *et al*, 2014; Reis *et al*, 2016). The recommended phase 2 dose (RP2D) of RG7388 administered orally was 600 mg BID for both the single agent and the combination arm. Six of 29 patients (21%) treated with single agent RG7388 achieved a CR or CRi/MLFS, while 11/46 patients (24%) treated with RG7388+cytarabine had a CR or CRi. Based on these data, a phase 3 trial of idasanutlin in combination with intermediate dose cytarabine in patients with refractory/relapsed AML is currently ongoing (NCT02545283). Interestingly, in those studies, pre-treatment MDM2 expression using flow cytometry correlated with clinical response suggestive of oncogene addiction. MDM2 expression may thus represent a valuable biomarker for the selection of AML patients who may be candidates for idasanutlin therapy (Reis *et al*, 2016). Safety and efficacy of another MDM2 inhibitor (MK-8242) in AML patients was reported recently. In this study, 26 patients with AML received MK-8242 orally twice daily. The maximum administered dose was 300 mg BID for 7 days followed by 14 days of rest. Of 24-efficacy evaluable patients, one had a CR. Unfortunately, the study, which was initially designed to assess the safety and efficacy of MK-8242 alone and in combination with cytarabine, was closed prematurely due to pipeline prioritisation (Ravandi *et al*, 2016). Other MDM2 inhibitors, such as HDM201 (Novartis Pharma, Basel, Switzerland), are currently in phase 1 trials in patients with AML, but no data have yet been reported. One of the potential limitations to the use of MDM2 inhibitors is the emergence of *TP53* mutations as a mechanism of resistance (Jung *et al*, 2016), while *TP53* mutant cells may also be selected for by cytotoxic chemotherapy (Wong *et al*, 2015).

## Targeting the BCL2 family in AML

*B-cell lymphoma 2* (*BCL2*) was discovered on B-cell leukaemia and follicular lymphomas more than 30 years ago (Fukuhara and Rowley, 1978; Tsujimoto *et al*, 1984). Although it was characterised as a *bonafide* oncogene, the mechanisms by which BCL-2 induces transformation (namely by blocking apoptosis) were only understood later. Within the following decade, a dozen of structurally related proteins were described. As introduced earlier, these proteins can be classified into three different groups: (i) the multidomain anti-apoptotic proteins such as BCL2, BCL-XL and MCL1 for example, (ii) the multidomain pro-apoptotic effector proteins such as BAX or BAK and (iii) the BH3-only group of pro-apoptotic proteins. This lalter group can be further subdivided into activator proteins, such as BIM, BID or PUMA, and sensitisers, such as BAD, BIK or NOXA for example (reviewed in Letai, 2008 and Hata *et al*, 2015). The major role of both BCL-2 and BCL-XL in promoting survival of cancer cells has made these proteins interesting targets for specific inhibition. MCL1 has also emerged as a mechanism of resistance to apoptosis and to BCL-2/BCL-XL inhibitors, and as such is considered a potential therapeutic target (Kotschy *et al*, 2016). So far, the number of BCL-2/BCL-XL inhibitors that have entered the clinic is limited and results in AML have been heterogeneous. Many compounds initially depicted as BH3-mimetic or BCL2 inhibitors have failed to show selectivity and actual binding to BCL2-related proteins in cells (van Delft *et al*, 2006; Soderquist and Eastman, 2016). However, several second-generation Bcl-2-specific inhibitors are currently in later stages of development in B-cell malignancies and venetoclax was recently approved for the treatment of patients with TP53 deleted chronic lymphocytic leukaemia.

Obatoclax, a first-generation pan-Bcl-2 family antagonist (putative BH3 mimetics) was assessed in phase I/II study in patients older than 70 years with treatment naive AML (Schimmer *et al*, 2014). The rational for this study was essentially based on the observed clinical responses in a few patients with AML treated in the phase I study (Schimmer *et al*, 2008). Obatoclax was initially given at 30 mg per day for 3 days, but the dose was reduced to 20 mg per day for 3 days due to neurological toxicity (confusion, somnolence and ataxia) in the first three patients. In the phase II part, patients received either 20 mg per day for 3 days or 60 mg over 24 h. None of the 18 patients who received obatoxclax achieved a CR. From the safety perspective, the most common adverse events were mild transient neurological effects such as euphoria, ataxia, somnolence and dizziness (Schimmer *et al*, 2014). This adverse effect profile was probably related to a lack of specificity of obatoclax and led to the termination of the programme.

Venetoclax (formerly ABT199), a second-generation, specific antagonist of Bcl-2 showed promising single agent activity in patient-derived AML samples (Pan *et al*, 2014). These results were confirmed in a recently published study. In this study, venetoclax was administered orally in 32 patients with AML at a dose of 800 mg daily (continuous dosing), with an intrapatient dose escalation during the first week, in order to mitigate the risk of tumour lysis syndrome (Konopleva *et al*, 2016). The majority of patients had previously received chemotherapy and/or a demethylating agent. The CR/CRi rate was 19% (6 of 32), and another six patients experience bone marrow blast reduction not reaching the threshold for CR (Konopleva *et al*, 2016). The majority of responses were seen within the first 4 weeks of therapy. However, the duration of complete or partial remission was limited, and all patients discontinued venetoclax due to disease progression. The most common adverse events seen with venetoclax were nausea, diarrhoea, and vomiting as well as febrile neutropenia. In a subsequent study, venetoclax was combined with azacytidine or decitabine in patients with treatment-naïve AML. In this ongoing study, 39 elderly (age⩾65) patients with intermediate or poor-risk cytogenetics not eligible for intensive induction therapy were treated with either decitabine (20 mg m^−^^2^, days 1–5) or azacytidine (75 mg m^−^^2^ days 1–7) combined with ascending doses of venetoclax daily (Pollyea *et al*, 2016). Doses of venetoclax up to 800 mg daily were well tolerated and produced a 76% overall response rate, which compares favourably with single agent demethylating agents, and some responses were durable. Based on these promising results a phase 3 trial is currently under way to compare venetoclax+azacytidine to single agent azacytidine in patients with treatment-naïve AML not eligible for intensive therapy (Table 1). In another phase 1b study, the feasibility and efficacy of venetoclax (600 mg per day) combined with low dose cytarabine (20 mg m^−^^2^ days 1–10 every 28 days) was assessed in 20 elderly patients with AML, some of them previously treated with demethylating agents. The combination was well tolerated and yielded an overall response rate of 75%. Based on these results a phase 3 trial is currently recruiting to compare venetoclax+low dose cytarabine to low dose cytarabine alone (Table 1). Other BCL2-specific inhibitors, such as S055746 are currently in phase 1 clinical trial (Table 1).

In preclinical models, MCL1 was shown to contribute to resistance to venetoclax in AML cells, and this could be overcome by DNA-damage-mediated reduction in MCL1 levels using standard of care cytotoxics such as daunorubicin and cytarabine (Niu *et al*, 2016). Although this provides a rational for combining BCL2 inhibitors with standard chemotherapy, overlapping toxicities of venetoclax and cytotoxic agents may limit the feasibility of combining these agents together. Several specific MCL1 inhibitors have recently entered clinical, but no data has yet been reported on their clinical activity (MIK665/S64315-NCT02992483 and NCT02979366; and AMG-176-NCT02675452).

## Targeting XIAP and IAP

Several inhibitors of various IAP have entered the clinic in the last decade with a couple being tested in AML or haematology-specific studies. From a pharmacological standpoint the first generation of drugs were in most cases antisense oligonucleotides while later generation agents are more often small molecule SMAC mimetics.

AEG35156 is an antisense oligonucleotide targeting XIAP. The rational for targeting XIAP is based on its potent caspase-inhibitory effect as well as correlative studies implicating XIAP in therapy resistance (Zhang *et al*, 2002; Tamm *et al*, 2004). In early phase studies performed in patients with relapsing/refractory patients, interesting results were seen with AEG35156 combined with idarubicin and high dose cytarabine as reinduction: among patients treated at the 350 mg m^−2^ dose level of AEG35156 15 of 32 (47%) reached a CR/CRp, the CR rate was 10/11 among patients with refractory AML (Schimmer *et al*, 2009). Based on these promising results, a randomised phase II trial was conducted comparing idarubicin (12 mg m^−2^ for 3 days) and cytarabine (1.5 g m^−2^ for 3 or 4 days depending on the patient’s age), with or without AEG35156 650 mg given intravenously on days 1, 2, 3 and 8 (Schimmer *et al*, 2011). However, the study was terminated early (after 41/60 patients were enrolled) when an interim analysis showed that the study would not meet its primary endpoint of increased CR rate (from 50 to 70%). In the final analysis, the CR/CRp rate was 41% in the experimental arm *vs* 69% in the control arm (*P*=0.18). Interestingly, although the addition of AEG35156 did not significantly alter the safety profile of the induction regimen, three patients died during the induction in the AEG35156 arm of the study while none of the 13 patients treated with conventional chemotherapy did (Schimmer *et al*, 2011).

Erba and colleagues reported on a study investigating LY2181308, a survivin antisense oligonucleotide as a single agent and combined with idarubicin and cytarabine in patients with refractory/relapsed AML (Erba *et al*, 2013). In both study arms, patients received a loading dose of 750 mg per day of LY2181308 for 3 days, given intravenously, followed by 750 mg every week. In the chemotherapy arm, patients received in addition idarubicin 12 mg m^−2^ per day and cytarabine 1.5 mg m^−2^ per day on days 4, 5 and 6. Chemotherapy was repeated every 28 days. Eight patients with high survivin expression (based on flow cytometry analysis at baseline) were treated in the single agent arm and no response was seen although exposure to LY2181308 did reduce survivin in peripheral blasts. In the combination arm, 4/16 (25%) achieved a CR, while a total of 9 patients (56%) were considered to have clinically meaningful response. Moreover, although LY2181308 was responsible for moderate myelosuppression, fatigue and flu-like syndrome (Tanioka *et al*, 2011) as a single agent, it did not appear to significantly alter the safety of the chemotherapy regimen used in this study (Erba *et al*, 2013). Although this was a small study with a heterogeneous patient population (all patients were previously treated and 10 patients had had prior transplant), LY2181308 and standard chemotherapy did not appear to lead to increased activity over what is expected from chemotherapy alone. There is currently no ongoing study with LY2181308 (www.clinicaltrials.gov accessed on 13 October 2016).

DiPersio *et al* recently reported on the activity of Debio-1143 combined with daunorubicin and cytarabine in patients with relapsed or poor risk AML, aged 75 years or less (DiPersio *et al*, 2015). Debio-1143 is an orally administered, monovalent SMAC-mimetic and binds preferentially to cIAP1 and cIAP2, and with less affinity to XIAP (Cai *et al*, 2011). The maximum tolerated dose of Debio-1143, administered orally on days 1–5, combined with the standard ‘7 plus 3 regimen’ was 400 mg given once daily. Although doses of 200 and 300 mg were shown to be safe, the 100 mg dose was selected for further studies due to apparent increased efficacy in the 100 mg cohort. A total of 11 patients achieved a CR, two patients had a CRi, while one patient had a partial response (overall response rate 14/29, 48%)(DiPersio *et al*, 2015). The most common adverse events related to treatment were nausea, diarrhoea and febrile neutropenia which is consistent with some of the side effects seen with single agent Debio1143 (nausea, vomiting and fatigue)(Hurwitz *et al*, 2015).

Birinapant (formerly TL32711) is a bivalent SMAC-mimetic that displays preferential binding to cIAP1 relative to cIAP2 and XIAP (Condon *et al*, 2014). In preclinical studies, birinapant showed potent anti-tumour activity alone and combined with various agents, including azacitidine, a demethylating agent, in models of AML (Carter *et al*, 2014). Preliminary data from a phase 1, dose and schedule optimisation study of birinapant were reported at the 2014 annual meeting of American Society of Hematology. In this study patients 20 with AML (*n*=19) and myelodysplastic syndrome (MDS) (*n*=1) were treated at various dose levels and schedules. Birinapant 17 mg m^−2^ twice a week appeared to be the optimal schedule in this early report. The best response to therapy was stable disease observed in some patients (Frey *et al*, 2014). The most common adverse events seen with birinapant include nausea, vomiting and diarrhoea, as well as fatigue, fever and cytokine release syndrome, which seems to be an on-target toxicity of IAP inhibitors. Bell’s palsy was also a dose-limiting toxicity with birinapant and was observed in patients with AML but also those with solid tumours treated in a separate study (Amaravadi *et al*, 2015), as well as those treated with other bivalent IAP inhibitors (Sikic *et al*, 2011), which suggests that it may be an on-target effect, although the mechanism remains uncertain. A study combining birinapant with azacitidine in patients with MDS and chronic myelomonogenous leukaemia was initiated based on the data reported by Carter *et al* (2014). However, this study was prematurely terminated when a preplanned interim analysis showed no increased efficacy for azacitidine+birinapant *vs* azacytidine alone (Donnellan *et al*, 2016). Interestingly, Brumatti *et al* recently showed that co-administration of a caspase inhibitor may increase birinapant-induced cell death by blocking caspase-8 and apoptosis, and inducing necroptosis (Brumatti *et al*, 2016). This opens the way for therapeutically targeting alternative cell death mechanisms such as necroptosis. The same group reported on the synergistic effect of combining a p38*α* inhibitor (such as LY2228820) with birinapant on AML both *in vitro* and *in vivo*, where the effect of the combination was this time dependent on TNF-induced apoptosis, and not necroptosis (Lalaoui *et al*, 2016).

Data regarding another inhibitor of SMAC mimetic (CUDC-427) were recently published (Tolcher *et al*, 2016), but no safety or efficacy data have yet been reported in patients with haematological malignancies. Likewise, initial safety data were reported for LCL161, another SMAC mimetic, as well as interesting data in combination with cyclophosphamide in patients with multiple myeloma but there is no ongoing study in patients with AML (Infante *et al*, 2014; Chesi *et al*, 2016).

## Targeting death receptors

Although death receptors are expressed in AML cells and some preclinical data indicate *in vitro* activity of DR4 agonist, there has been no clinical development of these drugs in AML, likely because of their collective lack of clinical activity in solid tumours. Newer compounds are currently being evaluated such as bispecific antibodies, but their relevance to AML is currently unknown (e.g. RO6874813, currently in phase 1 clinical trial). The recent observation that DR5 agonist may be able to downregulate myeloid-derived suppressor cells *in vivo* (Dominguez *et al*, 2017) may renew interest in death receptor agonists as potential combination partners with other immune modulating agents. Similarly, Chesi and colleagues recently showed that aside from their effect as inducers of apoptosis, SMAC mimetic (IAP antagonists) were able to induce anti-tumour immunity by modulating the NF-κB response in immune cells in the tumour microenvironment (Chesi *et al*, 2016). Together these data suggest that because the extrinsic apoptotic pathway regulates both apoptosis and the inflammatory response through NF-κB, drugs targeting this pathway (namely death receptor agonists and SMAC mimetics) may have immune modulatory properties that may allow their combination with other immune modulatory agents such as inhibitors of the immune checkpoints.

## Conclusion

Targeting the apoptosis machinery is a promising therapeutic approach in AML. In most cases, the goal is to eliminate leukaemic cells by reactivating cell death pathways, either by direct targeting of anti-apoptotic proteins such as those of the Bcl-2 or the IAP families, or by reactivating the p53 response. Most of the molecules targeting anti-apoptotic proteins that entered clinical trials demonstrated their tolerability, at least when tested alone, and in some cases when used in combination with cytotoxics. The full therapeutic activity of these molecules will probably be best realised through combination with other, cytotoxic or targeted, anticancer agents. Demethylating agents for example may be good mechanistic candidates for such approaches, because they induce the expression of death receptors in leukaemic cells (Karlic *et al*, 2014) but also sensitise leukaemic cells to IAP inhibitors (Carter *et al*, 2014), although the clinical relevance of this finding still remains to be shown.

Combinations of different drugs targeting apoptosis can also be envisaged. A recent study showed that the combination of an anti-MDM2 and a BET-inhibitor (targeting p53 and Myc) was able to eliminate leukaemic stem cells in a chronic myelogenous leukaemia model (Abraham *et al*, 2016). Other combinations are currently under clinical evaluation, including Bcl-2 inhibitors combined with MDM2 antagonists (Lehmann *et al*, 2016) or MEK inhibitors (Zhang *et al*, 2010) (Table 1). Apoptosis-inducing agents such as MDM2 or Bcl-2 antagonists may then represent an ‘apoptosis backbone’ for combination therapies in AML; however, the optimal combination partners remain to be determined. In their recently reported study, Gu and colleagues showed interesting activity of new MDM2 inhibitors that compromise XIAP mRNA translation by targeting the MDM2 RING domain, in an acute lymphoblastic leukaemia model (Gu *et al*, 2016). Taken together, these reports suggest that targeting simultaneously several nodes of apoptosis regulation may be more efficacious than targeting a single node. Also, emerging data suggest that alterations implicating alternative cell death mechanisms, such as autophagy and necroptosis for example, play a role in oncogenesis and tumour progression in various cancer models, including leukaemia. For example, autophagy which is an adaptive survival mechanism that allows the recycling of cellular constituents during conditions of cellular stress has been shown to contribute to leukaemia initiation and resistance to therapy, although this seems to be subtype specific (Auberger and Puissant, 2017). Similarly, necroptosis is a recently identified mechanism of regulated cell death.

One key question is the integration of these promising agents in the current therapeutic armamentarium. The only agent currently in late stage investigation is the MDM2 inhibitor idasanutlin (NCT02545283). This study compares cytarabine alone to cytarabine combined with idasanutlin, in first or second relapse, after failure of at least one conventional intensive induction regimen. Overall some overlapping toxicities of apoptosis targeting drugs with those of conventional cytotoxic agents may render their combination quite toxic or feasible only in young and fit patients. The feasibility of combining apoptosis targeting drugs with each other or with other targeted agents remains to be demonstrated in the clinic as is their relevance to clinical subgroups of AML patients. Indeed, many patients with AML may not be candidate to intensive induction chemotherapy because of older age or poor risk features. Overall, three settings can be envisioned: (1) combination with standard of care chemotherapy to improve percentage and/or duration or response for fit patients; (2) treatment of fit patients with chemotherapy-refractory disease and/or for patients with poor risk cytogenetics; this will probably involve combinations of apoptosis-targeting drugs with other targeted agents (demethylating agents for example); and (3) therapy for unfit patient, which will likely involve single agent regimens or carefully selected combinations.

The field of AML research is currently being revolutionised by the recently published molecular classifications (Patel *et al*, 2012; Cancer Genome Atlas Research Network, 2013; Papaemmanuil *et al*, 2016). It is tempting to speculate that these classifications may ultimately guide therapy for patients with AML, in a so-called ‘precision medicine’ approach. Recent examples including the development of FLT3 or IDH1-2 inhibitors illustrate this concept (Cortes *et al*, 2013; Hansen *et al*, 2014; Stein *et al*, 2014). However, a large proportion of patients harbour non-druggable genomic alterations. There is thus room for alternative strategies like the targeting of apoptosis that should be active across molecular classes of the disease. How molecular alterations will correlate with clinical activity of modulators of apoptosis is currently largely unknown. Of course, mutations in the *TP53* gene have been shown as a mechanism of both primary and secondary resistance to MDM2 inhibitors (Jeay *et al*, 2015), but the molecular alterations underpinning the activity of other inhibitors of anti-apoptotic proteins in AML remains to be identified. Currently, the expression of Bcl-2, MDM2 and XIAP seems to be the biomarker of choice for Bcl-2 inhibitors, MDM2 antagonists and XIAP inhibitors/SMAC-mimetics, but the expression of these proteins may vary at different stages of the disease (primary diagnosis *vs* relapse *vs* refractory setting) and how this correlates with the various subclasses of AML is still currently under investigation.

## Figures and Tables

**Figure 1:**
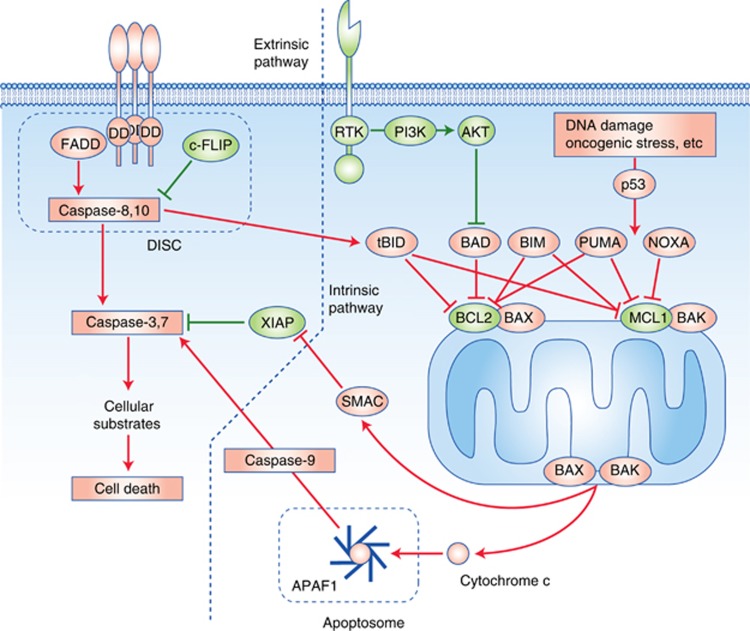
**Schematic representation of the intrinsic and extrinsic pathways of apoptosis.**

**Table 1:** Therapies targeting apoptosis currently in clinical development

**Compound**	**Drug class/targets**	**Study in AML**	**Design**	**Status**	***N*** **(AML)**	**CR/PR**	**Refs**
**MDM2 inhibitors**							
Idasanutlin	MDM2-p53	Phase 1b	Combination with cytarabine	Completed	76	22/75 (29%) CRc	Reis *et al*
		Phase 3	Combination with cytarabine	Ongoing			NCT02545283
		Phase 1b/2	Combination with venetoclax	Ongoing			NCT02670044
MK-8242	MDM2-p53	Phase 1b	Single agent and combination with cytarabine	Terminated		1/24 (4%) CRi, 1/24 (4%) PR	Ravandi *et al* (2016)
HDM201	MDM2-p53	Phase 1a	Single agent	Ongoing			NCT02143635
CGM097	MDM2-p53	No					
	SAR405838	MDM2-p53	No				
**BCL2 inhibitors**							
Obatoclax	pan-BCL2	Phase 1/2	Single agent	Completed	19	0/19, 3 patients had minor marrow response	Schimmer *et al* (2014)
Navitoclax	BCL2, BCLXL	No					
Venetoclax	BCL2	Phase 1a	Single agent	Completed	32	6/32 (19%) CR/CRi 6/32 (19%) PR. But short lasting	Konopleva *et al* (2016)
		Phase 1b	Combination with azacytidine	Ongoing	29	13/29 (45%) CR, 11 (38%) Cri, 2 (7%) PR (early report)	Pollyea *et al* (2016)
		Phase 3	Combination with azacytidine	Ongoing			NCT02993523
		Phase 1b	Combination with low dose cytarabine	Ongoing			
		Phase 3	Combination with low dose cytarabine	Ongoing			NCT03069352
		Phase 1b/2	Combination with idasanutlin	Ongoing			NCT02670044
		Combination with cobimetinib (MEK inhibitor)	On-going			NCT02670044	
S055746	BCL2	Phase 1a	Single agent	Ongoing			NCT02920541
S64315/MIK665	MCL1			Ongoing			NCT02979366 NCT02992483
**XIAP/IAP inhibitors**							
AEG35156	XIAP antisense	Phase 1b	Combination with idarubicin and cytarabine	Completed	56	16 CR (29%), 15/32 patients (47%) in the expansion phase	Schimmer *et al* (2009)
		Phase 2	Combination with idarubicin and cytarabine	Terminated	50	11/27 with AEG35156 and chemotherapy *vs* 9/13 with std chemotherapy	Schimmer *et al* (2011)
LY2181308	Survivin antisense	phase 1b	Combination with idarubicin and cytarabine	Completed	24	4/16 CR among patients treated with LY2181308 combined with chemotherapy	Erba *et al* (2013)
Debio1143	SMAC mimetic	Phase 1b	Combination with daunorubicin and cytarabine	Completed	29	11/29 CR, 3/29 CRp, 1/29PR	DiPersio *et al* (2015)
Biniparant	SMAC mimetic	Phase 1a	Single agent	Completed	20	no CR/PR	
		Phase 2	Combination with azacytidine	Terminated	NR	ORR 32% for azacytidine alone, 29% for Azacytidine+birinapant. More myelosuppression and fatal AEs in the birinapant arm	Donnellan *et al* (2016)

Abbreviations: AE=adverse event; AML=acute myeloid leukaemia; BCL2=B-cell lymphoma 2; CR=complete response; IAP=inhibitor of apoptosis proteins; MDM2=mouse double minute 2; PR=partial response; SMAC=second mitochondrial-derived activator of caspases; XIAP= X-linked inhibitor of apoptosis.
